# Genetically predicted green tea intake and the risk of arterial embolism and thrombosis

**DOI:** 10.3389/fmed.2023.1156254

**Published:** 2023-03-22

**Authors:** Lingmei Jia, Yali Chen, Chang Liu, Yinyin Luan, Min Jia

**Affiliations:** Cardiovascular Medicine Department, The Second Hospital of Hebei Medical University, Shijiazhuang, China

**Keywords:** green tea intake, arterial embolism and thrombosis, Mendelian randomization, ischemic heart disease, stroke, genome-wide association study

## Abstract

**Background:**

In previous observational studies, green tea intake has been demonstrated to protect against arterial embolism and thrombosis. However, whether there is a causative connection between green tea intake and arterial embolism and thrombosis is currently unclear.

**Methods:**

A two-sample Mendelian randomization (MR) study has been designed to explore whether there is a causal association between green tea intake and arterial embolism and thrombosis by acquiring exposure and outcome data from previously published research. Data from the MRC-IEU (data on green tea intake, 64,949 participants) consortium and the FinnGen project (data on arterial embolism and thrombosis, 278 cases of arterial thrombosis and 92,349 control participants) has been utilized to determine the causal impact of green tea intake on arterial embolism and thrombosis.

**Results:**

We found that genetically predicted green tea intake was causally associated with a lower risk of arterial embolism and thrombosis (IVW odds ratio [OR] per SD decrease in green tea intake = 0.92 [95% confidence interval, 0.85–0.99]; *p* = 0.032). Moreover, the sensitivity analysis (both MR Egger regression and weighted median) yielded comparable estimates but with low precision. No directional pleiotropic effect between green tea intake and arterial embolism and thrombosis was observed in both funnel plots and MR-Egger intercepts.

**Conclusions:**

Our study provided causal evidence that genetically predicted green tea intake may be a protective factor against arterial embolism and thrombosis.

## 1. Introduction

Arterial embolism and thrombosis is the formation of a blood clot inside an arterial blood vessel or the arterial thrombus coming from the heart, proximal arterial wall, or other sources, leading to the obstruction of blood flow in the arterial circulatory system, (1) acting as the cause of ischemic heart disease and stroke and the most common causes of death in the developing and developed countries (2, 3). Despite numerous advances in diagnosis and treatment, arterial embolism, and thrombosis remain a challenge for clinicians. Many lifestyle factors are involved in the occurrence and development of arterial embolism and thrombosis, such as diet, smoking, and physical inactivity (4). Therefore, the primary prevention for the risk of arterial thrombosis may be crucial.

The effects of green tea intake on reducing arterial thromboembolic risk have been proved by many observational studies. However, the findings and conclusions of those studies are partly contradictory. The results of a meta-analysis of observational evidence from prospective studies (5) revealed a reduced risk of coronary heart disease (CHD) associated with an increased green tea consumption of three cups per day (risk ratio 0.73, 95% CI 0.53–0.99), cardiac death (0.74, 95% CI 0.63–0.86), stroke (0.82, 95% CI 0.73–0.92), and mortality (0.76, 95% CI 0.63–0.91). Similarly, another meta-analysis, which enrolled both observational and randomized trials, showed a lower risk of stroke and myocardial infarction in patients with higher tea consumption (6). However, some other studies found that green tea intake indeed increases cardiovascular disease risk. Zahra Gaeini et al. suggested that each 1 cup/day increased habitual consumption of tea was related to a 4 and 14% increased risk of cardiovascular disease (7). Nevertheless, other unmeasured lifestyle components, like other underlying disorders, physical activity, or smoking habits, may have impacted these findings. Therefore, it remains unclear whether green tea consumption is causally associated with arterial embolism and thrombosis.

**Figure 1:**
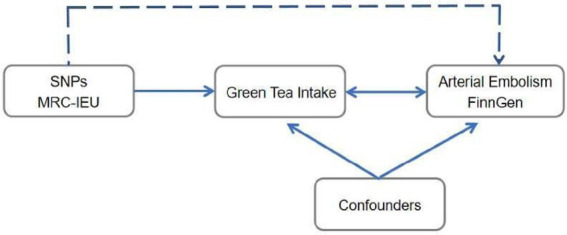
Schematic representation of our MR analysis.

Mendelian randomization (MR), (8) which employs genetic variants as instrumental variables, is an epidemiological approach that avoids potential confounders or reversing causality. According to Mendel’s second law, this method is not susceptible to reverse causality or confounding, analogous to a randomized trial where randomization to genotype occurs at conception. Consequently, MR is a powerful predictive tool for assessing causal associations, as shown in Figure 1 (9).

The SNPs associated with hypertension were selected from the MRC-IEU and the corresponding effect for these SNPs was estimated based on the effect of ED. MR is a powerfully predictive tool to test causal associations without any bias inherent to observational study designs due to the randomization and independence of alleles at meiosis.

In this study, through two-sample MR analysis, we investigated the causal association between green tea intake and arterial thrombosis using data from a genome-wide association study (GWAS) (10). We measured the association of 11 single nucleotide polymorphisms (SNPs) for green tea consumption with an arterial embolism and thrombosis study of 278 cases and 92,349 controls from the FinnGen project.

## 2. Methods

### 2.1. Overall study design

The MR data analyzed in this study were taken from our previous work and the institutional review board had given its approval to each of the published studies. Therefore, there was no need for further approval in the present study (11). Here, two-sample MR was used to investigate the causal association between gene-risk factors (e.g., Green tea intake) and gene outcome (e.g., arterial embolism and thrombosis) (12).

### 2.2. Data sources

#### 2.2.1. SNPs identification associated with green tea intake

The SNPs associated with green tea intake were identified from 64,949 individuals from the MRC-IEU Traits consortium based on the European populations (Output from GWAS pipeline using Phesant derived variables from UKBiobank), which was the most recent GWAS on green tea intake when we started the MR analysis. In the original GWAS study, the green tea intake was reported as a categorical ordered variable (cups of green tea) with no covariates considered in their model in the UK Biobank (data field: 100420). The summary statistics were generated following the exclusion of samples of poor quality and the original questionnaire divided the number of cups of tea into 0, 1, 2, 3, 4, 5, and ≥ 6 cups. As mentioned in the original study, one cup means 250 mL. A detailed description of the study design, including quality control procedures and statistical analyses, is available at http://www.nealelab.is/uk-biobank/.

#### 2.2.2. Study outcome: Arterial thrombosis

The SNPs of arterial embolism and thrombosis were obtained from the FinnGen project (FinnGen), which can be found at https://gwas.mrcieu.ac.uk/datasets/finn-a-I9_ARTEMBTHR. There were 278 cases of arterial thrombosis and 92,349 people who served as controls in this study, which has been permitted by their institutional review board, and all participants gave their informed consent as part of their original study.

### 2.3. Statistical analysis

We selected SNPs independent with a genome-wide significance, by following these criteria: First, the selected SNPs were significantly associated with green tea intake based on a genome-wide significance threshold (*p* < 5.0 × 10^−8^); (13). Second, SNPs in linkage disequilibrium with *r*^2^ < 0.001 and distance >10,000 kb were excluded (14, 15). Third, those SNPs weakly associated with instruction variants and green tea intake were excluded.

Due to no available individual-level GWAS data, the two-sample MR was used to estimate the effect of green tea on arterial thrombosis. Assumptions of MR are violated by horizontal pleiotropy (16), which is a mechanism by which genetic variants can influence the outcome rather than just exposure. Therefore, three methods (IVW, Weighted median, and MR Egger regression) were used as a safeguard against this in the present MR analysis (17).

Different horizontal pleiotropy models underlie each analytical approach. The consistency of all three methods can help us ensure that our conclusions are solid (18). The study’s statistical coding and related data are available from the corresponding author based on your reasonable request. R version 4.0.3 (2020-10-10; The R Foundation for Statistical Computing, Vienna, Austria) was used for all statistical analyses (19).

## 3. Results

### 3.1. Genetic factors for green tea consumption and arterial embolism and thrombosis

**Figure 2:**
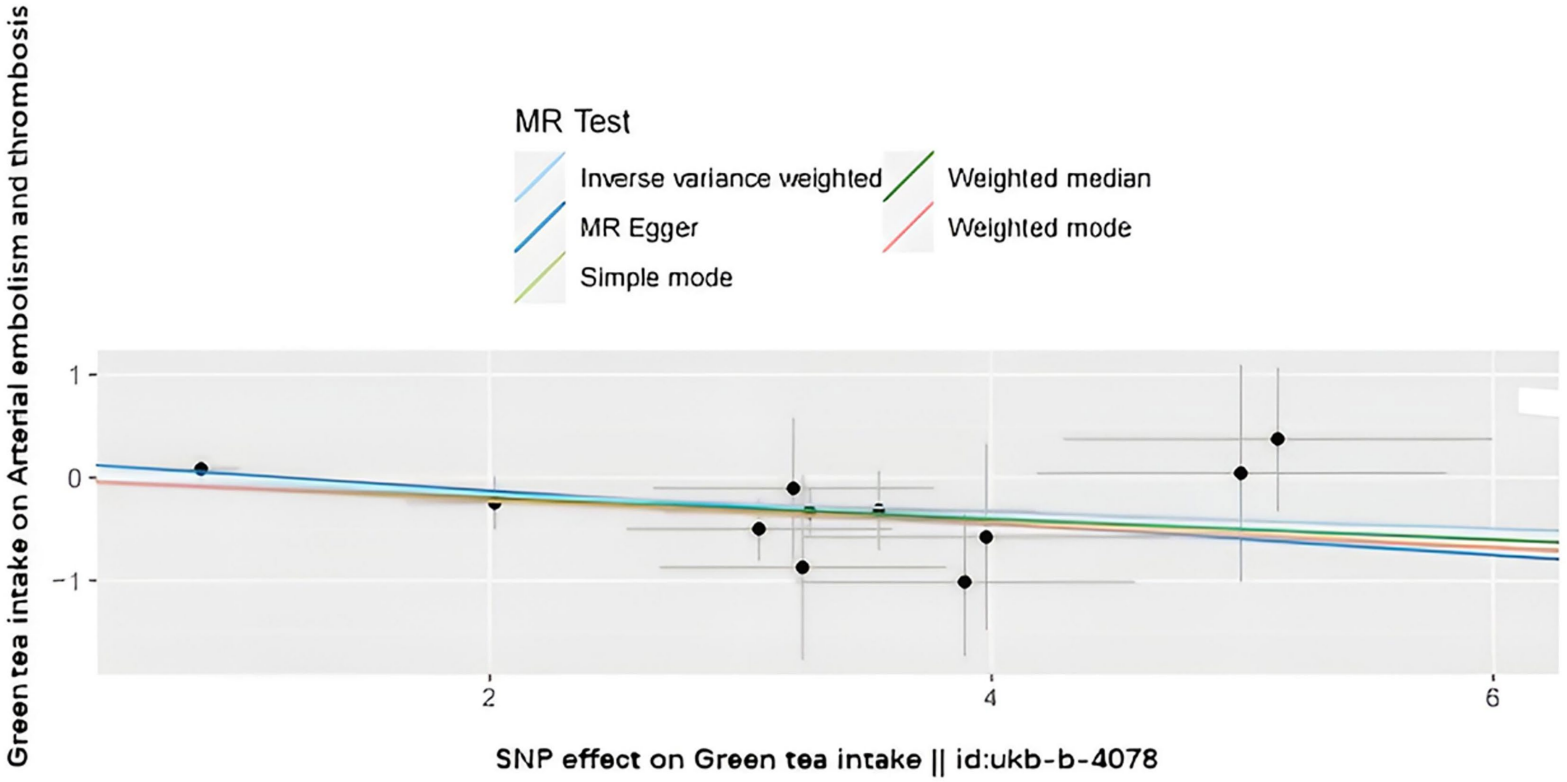
Scatter plot to visualize the causal effect of hypertension on the risk of ED. The slope of the straight line shows the magnitude of the causal association. IVW indicates inverse-variance weighted; and MR, Mendelian randomization.

**Figure 3:**
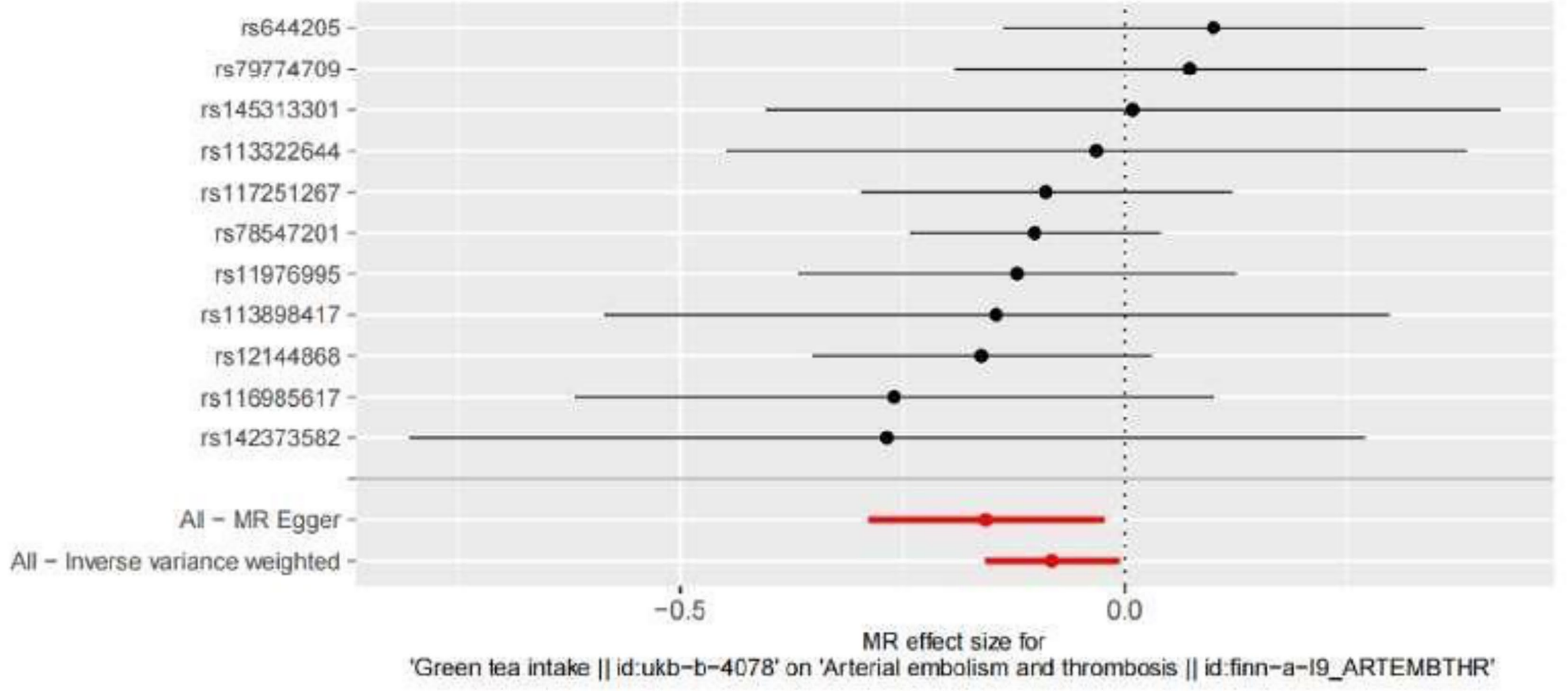
Forest plot to visualize the causal effect of every single SNP on ED. MR indicates Mendelian randomization.

The SNPs associated with green tea consumption and arterial embolism and thrombosis are summarized in Table 1. Eleven new genetic instruments had never been used in any previous research. The effects of the variant in green tea consumption on the risk of arterial embolism and thrombosis are shown in Figures 2, 3.

**Table 1:** List of genetic instruments.

SNP	Position	chr.	EA	OA	eaf.exposure	beta.exposure	se.exposure	*p* Value exposure
rs12144868	41,539,745	1	C	T	0.015146	3.07273	0.527463	5.70E−09
rs145313301	79,537,576	2	C	G	0.006149	4.99373	0.812513	7.90E−10
rs144954030	213,836,477	2	C	T	0.005665	4.88015	0.821066	2.80E−09
rs142811251	22,985,272	2	C	G	0.010296	3.45976	0.617814	2.10E−08
rs115952340	40,875,392	3	A	G	0.01334	3.00972	0.550266	4.50E−08
rs78547201	61,713,522	5	G	C	0.011607	3.27496	0.577735	1.40E−08
rs116985617	133,084,903	6	T	C	0.008531	3.89273	0.674419	7.80E−09
rs79774709	117,931,871	6	T	C	0.005782	5.14056	0.853785	1.70E−09
rs189140232	35,517,966	7	A	G	0.010006	3.56083	0.625652	1.30E−08
rs11976995	157,531,582	7	G	T	0.030423	2.01942	0.348689	7.00E−09
rs116035596	28,832,005	9	T	C	0.006559	4.90357	0.742882	4.10E−11
rs79638269	14,841,242	10	T	C	0.016328	2.67635	0.472895	1.50E−08
rs644205	89,745,814	10	A	G	0.196485	0.848874	0.150756	1.80E−08
rs183788045	64,006,281	11	T	G	0.008749	3.54822	0.64978	4.70E−08
rs117251267	95,673,607	13	C	T	0.01159	3.55014	0.617762	9.10E−09
rs142373582	104,757,781	14	C	T	0.014186	3.24633	0.565762	9.60E−09
rs113898417	21,614,895	16	G	C	0.006957	3.97893	0.726631	4.40E−08
rs62059726	48,715,705	17	A	G	0.015194	2.8216	0.511814	3.50E−08
rs12958992	71,259,772	18	G	A	0.026945	2.16091	0.378903	1.20E−08
rs117077082	42,928,868	20	G	A	0.031795	1.92055	0.34217	2.00E−08
rs113322644	33,992,170	21	A	C	0.011694	3.20963	0.555077	7.40E−09

SNP, single nucleotide polymorphism; EA, effect allele; OA, other allele; chr., chromosome; and se, standard error.

To evaluate the causal relationships between genetically predicted green tea consumption and arterial embolism and thrombosis, weighted median regression, IVW, and MR-Egger were utilized, as shown in Figures 2, 3. All three MR methods found broadly consistent support for a negative correlation between arterial thrombosis and consumption of green tea, suggesting that green tea consumption was causally associated with arterial embolism and thrombosis (IVW odds ratio = 0.92 per SD increase in green tea consumption [95% CI, 0.85–0.99]; *p* = 0.032). The MR Egger regression likewise yielded estimates that were also directionally similar (MR—Egger OR per SD increase in green tea consumption, 0.85 [95% CI, 0.75–0.98], *p* = 0.045).

### 3.2. Analysis of horizontal pleiotropy

**Figure 4:**
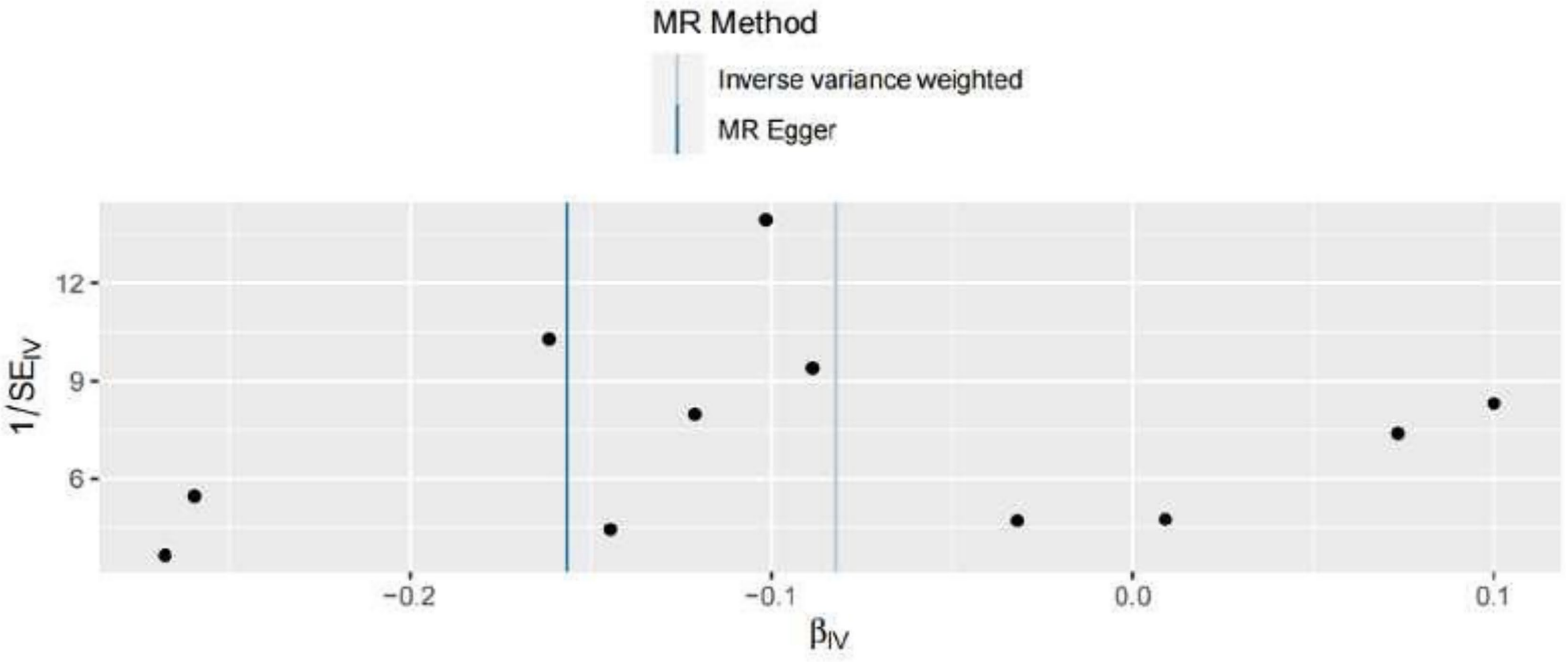
Funnel plots to visualize the overall heterogeneity of MR estimates for the effect of hypertension on the risk of ED. IVW indicates inverse-variance weighted, and MR indicates Mendelian randomization.

In the present MR analysis, the Funnel plot, MR-Egger intercept, and MR pleiotropy test were used for the detection of horizontal pleiotropy. The individual Wald ratios for every SNP plotted against their precision are displayed by Funnel plots, with asymmetry exhibiting directional horizontal pleiotropy. Nevertheless, it should be highlighted that assessing funnel plots for symmetry is challenging when only a few genetic instruments are utilized, as shown in Figure 4. In this research, there is no evidence of considerable directional pleiotropy for arterial thrombosis using the MR-Egger intercept and MR pleiotropy test (both *p* > 0.05), suggesting the association between green tea consumption and arterial embolism and thrombosis has no directional pleiotropic impacts.

### 3.3. Effects of individual genetic instruments on arterial embolism and thrombosis

**Figure 5:**
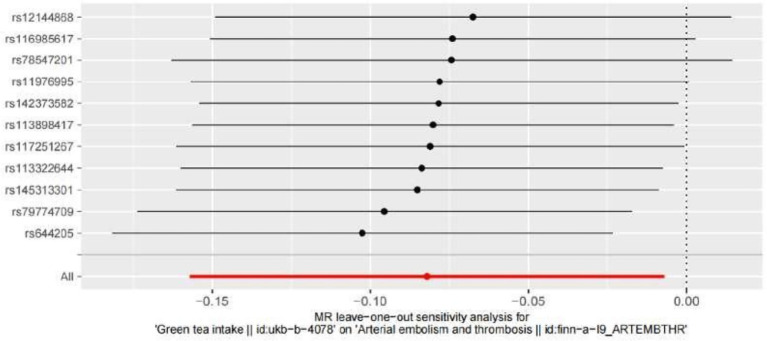
Leave-one-out plot to visualize the causal effect of hypertension on the risk of ED when leaving one SNP out MR indicates Mendelian randomization.

Leave-one-out analyses were carried out to see how each SNP affected the overall causal estimate. When individual SNPs were systematically eliminated and the MR analyses were performed again, no significant disparity in the estimated causal effect was observed, as shown in Figure 5. Thus, the projected effects could not be attributed to any single genetic instrument.

### 3.4. Three assumptions of MR analysis

Three other assumptions should be satisfied. Firstly, the instruction variants must be associated with green tea intake (Assumption 1). In the present MR analysis, we only SNPs with a genome-wide significance threshold (*p* < 5.0 × 10^–8^) to satisfy assumption 1. Secondly, the instruction variants affect the risk of arterial embolism and thrombosis only *via* green tea intake (Assumption 2). We performed MR Egger regression and no evidence of directional pleiotropic effects was observed in our study. Thirdly, confounders were not involved in the IVs, including the measured or unmeasured ones (Assumption 3), which was satisfied that both the exposure and outcome GWAS were finished in the European ancestry populations. Therefore, the heterogeneity of our population is relatively low.

## 4. Discussion

In the present study, we used an MR method to investigate the causal association between green tea intake and arterial embolism and thrombosis in a European ancestry population. Our results show that genetically determined green tea intake is causally associated with decreased arterial thromboembolic risk, which is in line with previous observational research. Our findings suggest that green tea intake may be a modifiable protective factor for arterial embolism and thrombosis.

Multiple studies have shown that lifestyle interventions are effective in reducing arterial thrombotic diseases, especially for high-risk individuals (16). Recently, the Framingham Offspring Study (20) found similar results: participants with intermediate or ideal cardiovascular health were 33% less likely to develop hypertension and 25% less likely to develop cardiovascular diseases than individuals who had poor cardiovascular health in the past 5 years (21). Therefore, lifestyle intervention plays an important role in reducing the risk of cardiovascular diseases and has become a hotspot for recent research.

The preventive function of green tea on arterial thrombosis has been proved by many observational studies (22). Pang et al. evaluated the association between green tea consumption and the risk of ischemic-related diseases, and a total of nine studies on Japanese were included in their meta-analysis (6). Individuals who did not consume green tea had a 19, 24, and 15% increased risk of cardiovascular diseases, intracerebral hemorrhage, and cerebral infarction when compared with those who consume one cup of green tea per day, respectively. In addition, individuals who drank 1–3 cups of green tea per day had a 19 and 36% reduced risk of myocardial infarction and stroke when compared to those who drank 1 cup/per day, respectively. Furthermore, individuals who drank ≥4 cups/day had a 32% reduced risk of myocardial infarction when compared with those who drank 1 cup/day. However, all of those studies are observational and these results may have been affected by other confounding factors.

Our MR analysis is a contribution to make up for this deficiency of observational studies on the relationship between green tea intake and arterial thrombosis, avoiding being affected by confounding or reverse causation (23). Therefore, in our present study, we used the two-sample MR method to find the causal associations based on the summary data from the biggest GWAS studies for green tea intake (*n* = 64,949) and arterial thrombosis (up to 278 arterial thrombosis cases and 92,349 control) in European ancestry people. In this sensitivity analysis, the directional pleiotropy was estimated using three distinct approaches: weighted median regression, IVW, and MR-Egger. The consistency of the three methodologies suggested that our results were credible. Since one’s genetic variations are stable over a lifetime, the results of MR symbolize a lifetime protective impact of high green tea consumption against arterial embolism and thrombosis. Myocardial infarction and stroke are the most important and severe clinical manifestations of arterial thrombosis. Hence, identifying the potential protective factor of arterial thrombosis may be more effective in lowering the risk of myocardial infarction and stroke recurrence as well as occurrence. Exploring the causal association between green tea consumption and arterial embolism and thrombosis, in other words, could be very useful for clinical and social purposes.

Several possible mechanisms may explain the protective effect of green tea intake on the risk of arterial embolism and thrombosis. Grassi et al. measured brachial artery flow mediated dilation (FMD) in healthy individuals, which showed that drinking tea can improve endothelial function, suggesting that endothelial dysfunction may play a crucial part in arterial thrombosis pathogenesis (24). In addition to arterial thrombosis, green tea intake also plays an important part in other cardiovascular diseases. Widlansky et al. reported that acute epi-gallocatechin-3-gallate (EGCG) supplementation can improve brachial artery FMD in patients with cardiovascular disease and green tea is rich in acute EGCG (25). Redford demonstrated that the KCNQ5 voltage-gated potassium channel contributes activation to vasodilation by green tea, leading to lower blood pressure (26). A meta-analysis by Yarmolinsky et al., focusing on randomized controlled trials of at least 8 weeks and aiming for secondary prevention of hypertension among prehypertensive or hypertensive persons, showed statistically significant reductions in blood pressure with green tea or tea extract consumption (27). In addition, green tea has positive biological activities against chronic diseases such as cancer, metabolic syndrome, and type 2 diabetes, antibacterial and antiviral activity, protection against UV radiation, an increase of bone mineral density, and antifibrotic and neuroprotective properties (28–31). We still need to further study the potential mechanism of green tea intake for reducing arterial thrombosis. Thrombosis is a complex condition that arises from the interplay of genetic and environmental factors. The contribution of green tea intake to thrombus formation may be mediated by its impact on environmental factors, in addition to genetic factors. Therefore, it is important to consider both genetic and environmental factors when exploring the potential impact of green tea intake on thrombosis.

The major strength of our study is that the two-sample MR design can avoid potential confounding factors (9). To meet the assumptions of the MR analysis, some important measures were taken: Only SNPs at genome-wide significant levels showing a valid association with green tea intake were employed in our MR analysis for ensuring the legitimate link among risk variables and SNPs (such as green tea intake in our research). In our study, only SNPs were used if they showed sufficient genome-wide significance in European populations. Hence, we assume that the potential confounders in this research are very low. For ensuring that SNPs merely impact arterial thrombosis from green tea consumption (no pleiotropic effects), weighted medium and MR Egger regression were carried out, and no indication of directional pleiotropic effects was found in this finding.

However, it should be pointed out that excessive green tea consumption may have adverse health effects, including on the kidneys. In the present study, we have included individuals with a wide range of green tea intake, up to six or more cups per day. While we did not observe any significant associations between green consumption and adverse health outcomes within this range. It should be noted that our findings should not be extrapolated to higher levels of green tea intake.

We just utilized sum-level statistics in our analysis, not individual-level data, which could be a limitation. Therefore, we will not be able to investigate the causal relationship among subgroups, for instance, men, women, or one cup per day and two cups per day. Besides, it is well known that the risk factors are various in different races and ethnicities (32). And Finns have a unique genetic structure and profile compared to other European populations. Therefore, the conclusion should be further reconfirmed by using outcome data from the latest GWAS on European ancestry. Most importantly, the OR may always exaggerate the size of the effect compared with relative risk (33). However, the incidence of arterial embolism and thrombosis was only 0.3% [278/(278 + 92,349)] in the included populations, which is much lower than 5%. Therefore, we consider the OR and relative risk to be approximately equal in this study.

## 5. Conclusion

In summary, our study found that genetically predicted green tea intake may be causally associated with a lower risk of arterial embolism and thrombosis. And the conclusion also needs to be further reconfirmed in the future.

## Data availability statement

The original contributions presented in the study are included in the article/supplementary material, further inquiries can be directed to the corresponding author.

## Ethics statement

Ethical approval was not provided for this study on human participants because the MR data analyzed in this study were taken from our previous work and the institutional review board had given its approval to each of the published studies. Therefore, there was no need for further approval in the present study. The patients/participants provided their written informed consent to participate in this study.

## Author contributions

MJ and YC: conceptualization. LJ: methodology, writing—original draft preparation, and writing—review and editing. YL: software. CL: validation. MJ: visualization and supervision. All authors contributed to the article and approved the submitted version.

## Conflict of interest

The authors declare that the research was conducted in the absence of any commercial or financial relationships that could be construed as a potential conflict of interest.

## Publisher’s note

All claims expressed in this article are solely those of the authors and do not necessarily represent those of their affiliated organizations, or those of the publisher, the editors and the reviewers. Any product that may be evaluated in this article, or claim that may be made by its manufacturer, is not guaranteed or endorsed by the publisher.

## Abbreviations

CHD: Coronary heart disease
MR: Mendelian randomization
GWAS: Genome-wide association study
SNP: Single-nucleotide polymorphisms
CI: Confidence interval
OR: Odds ratio
SD: Standard deviation
FMD: Flow-mediated dilation
EGCG: Epi-gallocatechin-3-gallate
